# Reversible Protein Capture and Release by Redox-Responsive Hydrogel in Microfluidics

**DOI:** 10.3390/polym14020267

**Published:** 2022-01-10

**Authors:** Chen Jiao, Franziska Obst, Martin Geisler, Yunjiao Che, Andreas Richter, Dietmar Appelhans, Jens Gaitzsch, Brigitte Voit

**Affiliations:** 1 Leibniz-Institut für Polymerforschung Dresden e.V., Hohe Straße 6, 01069 Dresden, Germany; jiao@ipfdd.de (C.J.); geisler@ipfdd.de (M.G.); yunjiao.che@gmail.com (Y.C.); applhans@ipfdd.de (D.A.); 2 Organische Chemie der Polymere, Technische Universität Dresden, Mommsenstraße 4, 01062 Dresden, Germany; 3 Institut für Halbleiter- und Mikrosystemtechnik, Technische Universität Dresden, Nöthnitzer Straße 64, 01187 Dresden, Germany; franziska.obst@tu-dresden.de (F.O.); andreas.richter7@tu-dresden.de (A.R.)

**Keywords:** microfluidics, disulfide bonds, redox-responsive, hydrogels, protein capture and release, swelling behaviors, mechanical properties

## Abstract

Stimuli-responsive hydrogels have a wide range of potential applications in microfluidics, which has drawn great attention. Double cross-linked hydrogels are very well suited for this application as they offer both stability and the required responsive behavior. Here, we report the integration of poly(*N*-isopropylacrylamide) (PNiPAAm) hydrogel with a permanent cross-linker (*N,N′*-methylenebisacrylamide, BIS) and a redox responsive reversible cross-linker (*N,N′*-bis(acryloyl)cystamine, BAC) into a microfluidic device through photopolymerization. Cleavage and re-formation of disulfide bonds introduced by BAC changed the cross-linking densities of the hydrogel dots, making them swell or shrink. Rheological measurements allowed for selecting hydrogels that withstand long-term shear forces present in microfluidic devices under continuous flow. Once implemented, the thiol-disulfide exchange allowed the hydrogel dots to successfully capture and release the protein bovine serum albumin (BSA). BSA was labeled with rhodamine B and functionalized with 2-(2-pyridyldithio)-ethylamine (PDA) to introduce disulfide bonds. The reversible capture and release of the protein reached an efficiency of 83.6% in release rate and could be repeated over 3 cycles within the microfluidic device. These results demonstrate that our redox-responsive hydrogel dots enable the dynamic capture and release of various different functionalized (macro)molecules (e.g., proteins and drugs) and have a great potential to be integrated into a lab-on-a-chip device for detection and/or delivery.

## 1. Introduction

Microfluidics is a technique for accurately processing and manipulating small amounts of fluid in a microscale device with channels ranging from tens to hundreds of micrometers [1]. Some of the main advantages of microfluidics are the low consumption of reagents, high repeatability, fast reaction rates, and accurate control of physical/chemical properties [1,2,3,4,5,6]. One application of microfluidics is the capture and release of biomolecules and drug delivery on the micrometer scale [7]. Key features of microfluidic capture and release devices are their small volumes, large specific surface areas and strong mass and heat transfer within the microchannels [8]. The specific surface can be increased even further when hydrogel dots are integrated into the microfluidic system. A high number of hydrogel dots in a microfluidic chamber reactor enlarge the specific surface area within the device. A hydrogel scaffold and their chemical units present an enhanced surface area for the fluidic components to interact with microfluidic compartments [9,10]. These compartments allow for the physical entrapment of proteins (e.g., enzymes) or a chemical reaction to induce the swelling of stimuli responsive hydrogels [11]. Despite the various advances from the last two decades, the challenge of constructing microfluidic systems with stimuli responsive hydrogels which are able to capture and release biomolecules based on chemical bonds rather than physical interactions still remains [12,13,14,15,16,17]. Microfluidic systems with integrated double cross-linked stimuli-responsive hydrogels which respond to chemical switches, can overcome this challenge and are the topic of the work presented here.

Stimuli-responsive hydrogels can shrink and swell through expelling and absorbing additional amount of water, triggered by different external stimuli such as temperature [18], light [19], redox potential [20,21], pH [22], salinity [23,24,25], or electric fields [26]. They have been of widespread interest in recent years due to their great potential in applications as biosensors [27,28], microfluidic systems [9,10,29], tissue scaffolds [30], cell culture [31], or drug release [32,33,34]. Within microfluidics, especially the thermally responsive poly(*N*-isopropylacrylamide) (PNiPAAm) hydrogel matrix is used for example as valves or micropumps [22,35,36,37,38]. There are, however, only few microfluidic devices containing hydrogels which exploit a redox reaction [7,39]. The disulfide bond is an extremely valuable redox-responsive functional group with high reactivity. It is well known that disulfide bonds can dissociate or re-form depending on the redox potential [40,41]. Additionally, disulfide bonds can react with thiol groups and make an exchange reaction [42]. Briefly, the thiolate, as a nucleophile, attacks the disulfide bond to form a new disulfide bond and produces a new thiol leaving group [43,44]. As thiol groups frequently occur in proteins and other biological samples, biological applications, such as chemosensors and nanomaterial carriers based on the functionalization of disulfides have been developed [40,45,46].

**Figure 1:**
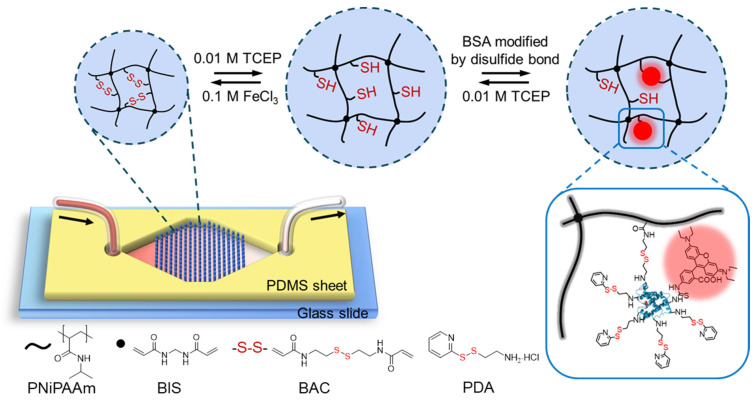
Schematic drawing of the microfluidic chip design and the reversible redox-responsive property of double cross-linked PNiPAAm hydrogel dots permanently cross-linked by BIS and reversibly cross-linked by the disulfide bonds of BAC. By the cleavage and re-formation of disulfide bonds in hydrogel dots, the capture and release of modified BSA can be achieved. BSA was modified with rhodamine B and PDA (bottom right), which introduced the disulfide bonds.

The introduction of disulfide bonds into hydrogels enables the formation of redox responsive systems [47,48,49] but also allows for the application of double cross-linked hydrogels containing a mixture of permanent and reversible cross-linking points. Combined with the commonly used *N,N′*-methylenebisacrylamide (BIS, Figure 1) as permanent cross-linker, the *N,N′*-bis(acryloyl)cystamine (BAC, Figure 1) is a very convenient disulfide-bearing redox responsive reversible cross-linker [50]. The double-crosslinked hydrogel system then shows an additional reversible swelling in response to changes in the redox potential while exposing thiols in the swollen state. When applied with P(NiP)AAm as a backbone, this BAC/BIS system has been reported and characterized for its viscoelastic properties as well as their ability to capture and release organophosphates [51,52,53]. Recently, our group also investigated the correlation between the mechanical properties, swelling behaviors and swelling kinetics of PNiPAAm-BAC-BIS hydrogels. Long-term cycle stability was achieved in bulk hydrogel and micro-structured hydrogel was realized by photopolymerization, laying a solid foundation for the application in microfluidics [54]. In order to integrate such a hydrogel into the microfluidic device, PDMS-on-glass device formed by a polydimethylsiloxane (PDMS) sheet and a glass holder has been developed [55,56,57]. PDMS can be shaped quickly by lithography, and fluid flow can easily be observed due to the high transparency of both PDMS sheet and glass slide [9,58]. In order to integrate a large number of hydrogels into a microfluidic device, the reported synthesis of an array of hydrogel dots proved to be a feasible platform [10,59].

Here, we report a simple and fast method to reversibly capture and release proteins using redox responsive hydrogels within a microfluidic device. Integration of PNiPAAm-BAC-BIS hydrogel dots into a microfluidic device was achieved by in-situ photopolymerization (Figure 1). Cleavage or re-formation of redox-responsive disulfide bonds changes the cross-linking densities of the hydrogel dots, making them swell or shrink in the microfluidic devices. The hydrogel dots were to capture and release the protein bovine serum albumin (BSA) modified with 2-(2-pyridyldithio)-ethylamine (PDA, source of disulfide bonds) and rhodamine B (RhB) via the thiol-disulfide exchange. Ideally, the protein capture and release could even be repeated over various cycles. Such a reproducible capture and release of a functionalized protein in microfluidics has great potential to be integrated in a lab-on-a-chip device for enzyme immobilization, rapid detection devices and/or delivery of captured macro (molecules).

## 2. Materials and Methods

### 2.1. Materials

*N*-isopropylacrylamide (NiPAAm, ≥99%), *N,N′*-methylenebisacrylamide (BIS, 99%), lithium phenyl-2,4,6-trimethylbenzoylphosphinate (LAP, ≥95%), tris(2-carboxyethyl)phosphine hydrochloride (TCEP), dimethyl sulfoxide (DMSO), cystamine hydrochloride (≥98%), 2,2′-dipyridyl disulfide (98%), iron(III) chloride hexahydrate (≥99%), bovine serum albumin (BSA, ≥96%), rhodamine B isothiocyanate (RhB), phosphate buffered saline tablet (PBS), *N*-ethyl-*N′*-(3-dimethylaminopropyl)carbodiimide hydrochloride (EDAC, ≥99%), 2-(*N*-morpholino)ethanesulfonic acid (MES, ≥99%), sodium peroxodisulfate (99%), *N,N,N′,N′*-tetramethylethylenediamine (TMEDA, 99%) were purchased from Sigma-Aldrich (Damstadt, Germany). *N,N′*-Bis(acryloyl)cystamine (BAC, 98%) was purchased from Alfa Aesar (Kandel, Germany). Sodium bicarbonate (99.5%) was purchased from ACROS Organics (Geel, Belgium). Sodium carbonate anhydrous (≥99.5%) was purchased from Honeywell Fluka (Seelze, Germany). Ethanol absolute (≥99.5%) was purchased from VWR Chemicals BDH (London, UK). Silicone elastomer kit (PDMS) was purchased from DOW Corning (Midland, MI, USA). Deionized water was used for all experiments. All chemicals were used as received without further purification.

### 2.2. Synthesis of 2-(2-pyridyldithio)-ethylamine (PDA)

PDA was produced following a published procedure [60]. Aldrithiol (1.1 g, 5.0 mmol, 2 eq.) was dissolved in 10 mL of methanol in a round bottom flask and 400 µL acetic acid was added. Then the cysteamine hydrochloride (0.28 g, 2.5 mmol, 1 eq.) dissolved in 5 mL of methanol was added dropwise in 30 min. The reaction was performed under argon atmosphere. After stirring for 48 h, the solvent was evaporated from the reaction mixture and the residue was purified by precipitation in cold diethyl ether overnight followed by filtration (2 times). The product was dried overnight in an oven at 40 °C. Yield: 0.42 g, 75%. The ^1^H NMR spectral data are consistent with the structure (Figure S1) in Supplementary Material. ^1^H NMR (500 MHz, D_2_O, *δ*): 8.47 (d, ^3^*J*_HH_ = 4.2 Hz, 1H, Ar H), 7.86 (td, ^3^*J*_HH_ = 7.7 Hz, ^4^*J*_HH_ = 1.8 Hz, 1H, Ar H), 7.76 (d, ^3^*J*_HH_ = 8.1 Hz, 1H, Ar H), 7.35 (td, ^3^*J*_HH_ = 6.2 Hz, ^4^*J*_HH_ = 1.0 Hz, 1H, Ar H), 3.37 (t, ^3^*J*_HH_ = 6.3 Hz, 2H, CH_2_C*H*_2_NH_2_), 3.13 (t, ^3^*J*_HH_ = 6.3 Hz, 2H, SC*H*_2_CH_2_).

### 2.3. Synthesis of BSA-RhB

BSA (500 mg, 7.6 μmol, 1 eq.) was dissolved in 1 mM carbonate buffer (25 mL, pH 10) and stirred for 30 min before adding RhB (6.1 mg, 11.4 μmol, 1.5 eq.) dissolved in 1 mL DMSO. After 20 h of stirring at room temperature, the mixture was extensively dialyzed against 1 mM PBS buffer (pH 7.4) for 3 days to remove all unbound RhB. All processes were performed under light protection. Finally, the purified mixture was freeze-dried overnight to get the product BSA-RhB, which was stored at −20 °C. Molecular weight was confirmed by matrix assisted laser desorption ionization–time of flight mass spectrometry (MALDI-TOF-MS) (*M*_BSA_: 66,400 Da and *M*_BSA-RhB_: 66,600 Da).

### 2.4. Synthesis of BSA-RhB-PDA

BSA-RhB (400 mg, 6.0 μmol, 1 eq.) and EDAC (57.7 mg, 300.0 μmol, 50 eq.) were dissolved in 50 mM MES buffer (25 mL, pH 6.5) and stirred for 30 min before adding dropwise PDA (13.4 mg, 6 μmol, 10 eq.) dissolved in 50 mM MES buffer (5 mL, pH 6.5). After 20 h of stirring at room temperature, the mixture was extensively dialyzed against 1 mM PBS buffer (pH 7.4) for 3 days to remove all unbounded molecules. All processes were performed under light protection. Finally, the purified mixture was freeze-dried overnight to get the product BSA-RhB-PDA, which was stored at −20 °C. Molecular weight was confirmed by MALDI-TOF-MS (details in part 2.7) (*M*_BSA-RhB-PDA_: 67,600 Da).

### 2.5. Preparation of Hydrogel Arrays

Photomask for patterning (array with diameter 350 µm, Figure S2a in Supplementary Material) was designed with the CAD 2021 software Autodesk Inventor (San Rafael, CA, USA) and produced on a black and white flat film by photo plotting (MIVA 26100 ReSolution, MIVA Technologies, Schönaich, Germany). Polyoxymethylene (POM) mold with single chamber (Figure S2c in Supplementary Material) used for the photopolymerization of gel precursor was produced in-house by milling on a four-axis CNC milling machine (DMU 50, DMG MORI, Bielefeld, Germany). The depth of the chamber was 151 ± 1 μm, confirmed by the confocal microscope (μsurf explorer, Nano Focus, Oberhausen, Germany). Before the photopolymerization of hydrogel arrays, the Menzel glass slide (76 mm × 26 mm × 1 mm) was cleaned with isopropanol, MilliQ and ethanol sequentially in an ultrasonic bath.

For the preparation of hydrogel precursor solution, the cross-linker BAC was first dissolved in ethanol and stirred for 20 min, then the monomer NiPAAm (930.8 mg, 12.5 wt %) and the other cross-linker BIS dissolved in deionized water were added. The entire amount of cross-linker is fixed at 2 mol% and with molar ratios (BAC:BIS) of 1:1, 1.5:1, 2:1, 3:1, 4:1, and 5:1, which were named as N 1:1, N 1.5:1, N 2:1, N 3:1, N 4:1, and N 5:1 hydrogel, respectively (Table S1). In order to dissolve all the cross-linkers, a higher amount of ethanol was used in the monomer solutions with a higher BAC ratio (0.71 mL ethanol used for N 1:1, N 1.5:1, N 2:1 hydrogels and 1.22 mL ethanol used for N 3:1, N 4:1, N 5:1 hydrogels). The amount of photoinitiator is 0.65 mol% to the monomer, which was added under light protection. The precursor solutions were purged with argon for 15 min to remove oxygen.

The preparation of the hydrogel dot arrays was divided into five steps (Figure S3 in Supplementary Material) and derived from a previously published procedure [10]. Firstly, the hydrogel precursor solution was transferred to the reaction chamber of the POM mold. Then, the washed glass slide was aligned on the chamber and patterned photomask was aligned on the glass slide. According to the design principle, an even layer without any trapped air was formed between the glass slide and the reaction chamber of POM mold. Afterwards, the reaction chamber was exposed to an UV lamp (DELOLUX 04, DELO, Windach, Germany) with a light power of 8 mW cm^−2^ on the sample surface. The emission spectrum for the photopolymerization ranges from 315 to 500 nm and the irradiation time is 7 s. Next, the glass slide with the covalently attached cylinder-shaped hydrogel dots was separated from the POM mould and immersed in the deionized water overnight to remove all the unbound precursor solution. After the as-prepared hydrogel dots reached equilibrium in deionized water, dried and then wiped the glass slide with isopropanol without touching the hydrogel dots. Finally, the microfluidic chip was sealed by aligning the glass slide on the PDMS sheet.

### 2.6. Microfluidic Testing

Reducing agent used was 0.01 M TCEP aqueous solution and oxidant used was 0.1 M FeCl_3_ aqueous solution. By purging different solutions (0.01 M TECP aqueous solution, 0.1 M FeCl_3_ aqueous solution) with different flow rate (1, 2, 5, 10, 50 µL min^−1^) through the hydrogel arrays with different hydrogel contents (N 1:1 to N 5:1 hydrogels) in the microfluidic chip, the swelling behavior of the microstructured hydrogel dots was observed by an optical microscope (Leica S8APO, DFC295 camera, cold light source: KL 1500 LCD). The swelling ratio (*S*_R_) of the hydrogel dots was calculated by Formula (1):

(1) $$S_{R}{=D}_{t}/D_{0}$$

where *D*_t_ and *D*_0_ represent pending test diameter and original diameter of the hydrogel dot, respectively.

For protein capture and release test, 0.01 M TCEP aqueous solution was first purged at the flow rate of 1 μL min^−1^ for 60 min, followed by deionized water washing for 30 min. Afterwards, 50 μM BSA-RhB-PDA aqueous solution was purged at the flow rate of 1 μL min^−1^ for 90 min, followed by 2 mM PBS buffer (pH 7.4) washing for 40 min. For protein release, 0.01 M TCEP aqueous solution was purged at the flow rate of 2 μL min^−1^ overnight. The described cyclic capture and release process of protein was repeated three times. The whole processes were observed by confocal laser fluorescence microscope (CLSM, Leica SP5, Wetzlar, Germany) at the height of 80 μm under both laser field (*λ*_excitation_: 561 nm, laser intensity: 15%) and bright field. The release ratios of protein were analyzed by ImageJ. One was purging 50 μM BSA-RhB-PDA aqueous solution at the flow rate of 1 μL min^−1^ for 90 min, followed by 2 mM PBS buffer (pH 7.4) washing for 40 min. The other was consistent with the purging experiment of the protein capture, except that BSA-RhB-PDA is replaced by BSA-RhB. The flow rate of all the chip washing was 5 μL min^−1^.

### 2.7. MALDI-TOF

The MALDI-TOF mass spectra were measured in linear positive detection mode by an autoflex^®^ speed MALDI-TOF system (Bruker Daltonics GmbH, Bremen, Germany) equipped with a smartbeam™ II (modified Nd:YAG) laser having a wavelength of 355nm. Sinapidic acid and *α*-cyano-4-hydroxycinnamic acid (HCCA) (both by Sigma Aldrich, Damstadt, Germany) were used as matrix (both 10 g L^−1^) dissolved in Methanol and mixed at a ratio of 1:1 (v/v). 1 µL of a 0.5 g L^−1^ methanolic sodium trifluoroacetate solution was added per 100 µL matrix solution. The sample was prepared in deionized water at a concentration of 2 g L^−1^, mixed with the matrix solution in a ratio of 1:1 (v/v) and spotted on the MALDI plate via the dried droplet method [61]. BSA of different batches was used for calibration and for verification. The samples were measured with an acceleration voltage of 19.5 kV, a laser attenuation of 30%, a laser repetition rate of 1 kHz and a detector gain of 70× (3.446 kV). Each mass spectrum was recorded by accumulation of 8000 shots.

### 2.8. NMR Measurement

^1^H NMR spectra were recorded on the Bruker Advance III spectrometer (Bruker Biospin, Ettlingen, Germany) at 500 MHz using deuterium oxide as the solvent. Chemical shifts of ^1^H NMR were referred to TMS (*δ* = 0).

## 3. Results and Discussion

### 3.1. Experimental Design and Microfluidic Chip Design

Precisely patterned cylinder hydrogel dot arrays with a diameter of 350 μm per dot were prepared through fast and efficient photopolymerization. Their height of 150 μm was determined by the POM mold (Figure S2) used for the polymerization. The preparation process and formation mechanism of PNiPAAm-based hydrogels are illustrated in Figure S3. Based on previous results, the re-oxidation of disulfide bonds in the double cross-linked hydrogel dots was based on a protocol for BAC/BIS double cross-linked macrogels [54]. 0.01 M TCEP aqueous solution has been reported to sufficiently reduce the disulfide bonds into thiol groups, and 0.1 M FeCl_3_ aqueous solution was proven to oxidize thiol groups into disulfide bonds. The redox-responsive PNiPAAm hydrogels were hence ready to be transferred into a microfluidic chip. For this, a single-chamber microfluidic chip with a chamber width of 14 mm was selected and prepared for the capture of proteins. Based on previously published considerations, a conical widening inlet and conical narrowing outlet was designed with an opening angle of 58° (Figure 1) to lead to a chamber containing the hexagonally arranged hydrogel dots [10]. Removing any trapped air was achieved by a freeze-pump-thaw process and an integrated bubble trap in the microfluidic set up (Figure S4) before purging the water into the chip.

### 3.2. Optimizing the Cross-Linker Composition of the Hydrogel

**Figure 2:**
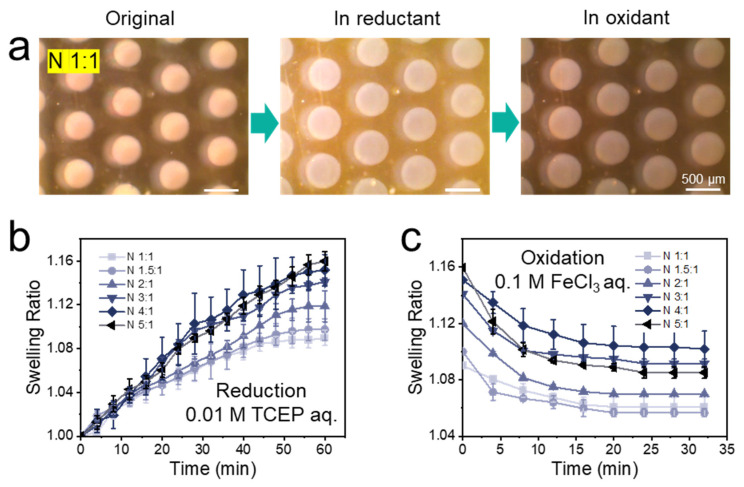
(**a**) Optical images of the original, the reduced (FR 1 µL min^−1^) and the oxidized (FR 2 µL min^−1^) N 1:1 hydrogel dots. (**b**) Swelling of PNiPAAm hydrogel dots with different molar ratios of cross-linkers BAC to BIS (from N 1:1 to N 5:1 hydrogel) in the microfluidic chip when 0.01 M TCEP aqueous solution perfused. (**c**) Shrinking of PNiPAAm hydrogel dots with different molar ratios of cross-linker BAC to BIS (from N 1:1 to N 5:1 hydrogel) in the microfluidic chip when 0.1 M FeCl_3_ aqueous solution was purged. At least three hydrogel dot arrays were tested for each experimental point to obtain reliable data. Longer time for oxidation process did not result in larger degree on shrinking for hydrogel dots.

The swelling ratio of hydrogel dots was expected to differ with different molar ratios of BAC to BIS. Thus, several molar ratios were tested (1:1, 1.5:1, 2:1, 3:1, 4:1, and 5:1) while keeping the overall amount of cross-linker constant at 2 mol% with respect to the monomer. All gels were named accordingly as N 1:1, N 1.5:1, N 2:1, N 3:1, N 4:1 and N 5:1. Kinetic measurements to note the swelling and de-swelling behaviors over time are shown in Figure 2b,c, respectively. TCEP and FeCl_3_ aqueous solutions were purged at a flow rate of 1 and 2 µL min^−1^ in the microfluidic chip, respectively. Photos of the original, the reduced and the oxidized N 1:1 (lowest amount of responsive cross-linker) hydrogel dots are shown in Figure 2a, while the ones of N 5:1 (highest amount of responsive cross-linker) hydrogel dots are shown in Figure S5. Irrespective of the different molar ratios of BAC to BIS, the degree of swelling and deswelling remained constant after 60 min of reduction and 20 min of oxidation. All final swelling ratios of the hydrogel dots, determined by measuring the diameters of the dots, where thus validated after 60 min of reduction and 30 min of oxidation (summarized in Table 1). With the increase of BAC content, the swelling ratio of hydrogel dots in reducing agent increased from 1.09 of the N 1:1 hydrogel to 1.16 of the N 5:1 hydrogel. The oxidative shrinkage rose from 33% of the N 1:1 hydrogel to 44% of the N 5:1 hydrogel, except that N 3:1 and N 4:1 hydrogels were around 34%. This meant that with increasing of BAC content, more disulfide bonds were cleaved by TCEP reduction. Thus, a higher decrease in cross-linking density is given which leads to more water absorption and higher swelling ratios. Similarly, the presence of more thiol groups in reduced hydrogel dots also gave a higher ratio of reformed disulfide bonds and hence a higher shrinking during the oxidation. No hydrogel returned to the original size since the hydrogels deswelled to the thermodynamically favored state in water, at which no complete disulfide reformation is assumed under the given experimental conditions. Thus, the ideal BAC/BIS ratio could be identified with these experiments.

In addition to the BAC content, the influence of different flow rates on swelling ratios of hydrogel dots has been studied. Kinetics for the swelling ratios of N 1:1, N 1.5:1, and N 2:1 hydrogel dots under 0.01 M TCEP purged at flow rate of 1, 10, and 50 µL min^−1^, respectively, are shown Figure S6. The hydrogel dots had a relatively low equilibrium swelling ratio (1.09 for N 2:1) at a high flow rate of 50 µL min^−1^, while there was no significant difference between 1 and 10 µL min^−1^ (both are 1.12 for N 2:1). This behavior indicated that high flow rates could prevent a complete reduction of disulfide units and hence a complete swelling of the hydrogel dots. In the follow-up microfluidic tests, a flow rate below 10 µL min^−1^ (1–5 µL min^−1^) was chosen to ensure a complete swelling.

### 3.3. Mechanical Properties of Bulk Hydrogels

For long-term experiments, the resistance of the hydrogels to the fluid shear force and their ability to maintain their shape were particularly important. Therefore, the mechanical properties of the bulk hydrogels were studied.

**Figure 3:**
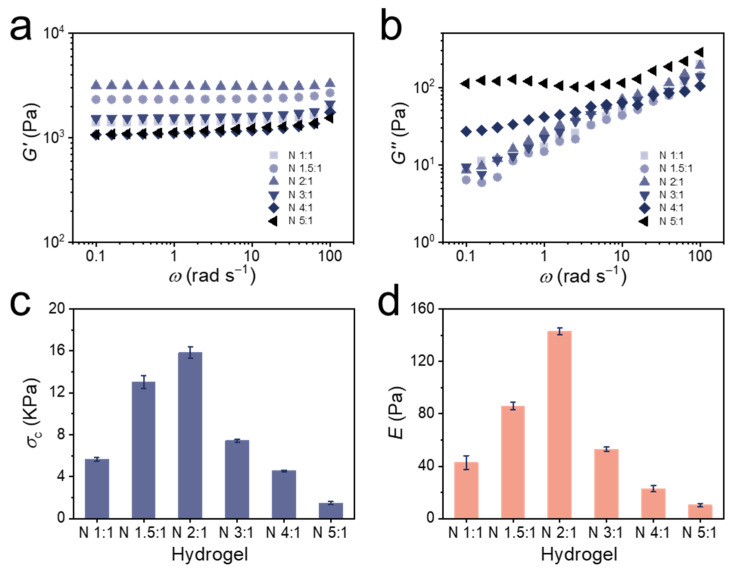
Frequency dependence of (**a**) storage modulus (*G′*) and (**b**) loss modulus (*G″*) for the bulk PNiPAAm hydrogel (from N 1:1 to N 5:1 hydrogel) at a fixed temperature (*T* = 25 °C) and strain (*γ* = 1%). (**c**) Compression strengths at 50% strain and (**d**) elastic moduli (*E*) of bulk PNiPAAm hydrogels (from N 1:1 to N 5:1 hydrogel). At least three bulk hydrogels were tested for each experimental point to obtain reliable data.

The frequency dependence of storage modulus (*G′*, Figure 3a) and loss modulus (*G″*, Figure 3b) was determined from all hydrogels alongside the loss factor (tan *δ*, Figure S7a). All hydrogels exhibited weak frequency-dependent viscoelastic behaviors. It was obvious that *G′* was always larger than *G″* in all the hydrogels, which was also reflected in that the tan *δ* is always much lower than 1, suggesting an elastic and fully cross-linked nature of the hydrogels [62,63]. Since the tan *δ* of an ideal covalent gel is zero, meaning all network chains sustain the stress, the extremely low tan *δ* value of the hydrogel indicated that there were few defects in the hydrogels [64,65]. *G′* generally remained constant, while *G″* increased with increasing frequency, which could be attributed to the cleavage of disulfide bonds at high frequencies. With increasing BAC content, the hydrogels possessed more reversible disulfide bonds that could be broken, resulting in higher *G″*. The latter became more pronounced at lower frequencies for N 4:1 and N 5:1, suggesting that these hydrogels were less feasible for an application in microfluidics.

In addition to rheological tests, compression tests also showed the same conclusion. The typical compression stress–strain (*σ*_c_–*ε*_t_, Figure S7b) curves of the bulk hydrogels were used to determine the compression strengths of 50% strain (Figure 3c) and elastic moduli (*E*, Figure 3d) were calculated from these measurements. All values are also summarized in Table 1. With the increasing BAC ratio, the compressive strength at 50% strain initially increased from 5.66 kPa (N 1:1) to 15.85 kPa (N 2:1), and then dropped over 7.43 kPa (N 3:1) to 1.47 kPa (N 5:1). Similarly, the elastic modulus of hydrogels increased from 43 Pa (N 1:1) to 143 Pa (N 2:1), and then dropped over 53 Pa (N 3:1) to 10 Pa (N 5:1). This behavior indicated that when subjected to external stress, the disulfide bonds broke and effectively dissipated the energy [66,67]. Thus, hydrogels with a higher BAC ratio first showed a higher compressive strength at 50% strain and elastic modulus until N 2:1. However, when the BAC:BIS ratio was higher than 2:1, the reduced amount of permanent cross-linker could not maintain the hydrogel network, and the large amount of reversible cross-linker then accelerated the fracture of the network. As a result, the elastic modulus of the N 3:1, N 4:1, and N 5:1 hydrogel dropped sharply and the hydrogel N 2:1 has proven to be the ideal candidate to be used in microfluidics due to the most suitable mechanical properties.

### 3.4. Modifications of BSA

**Figure 4:**
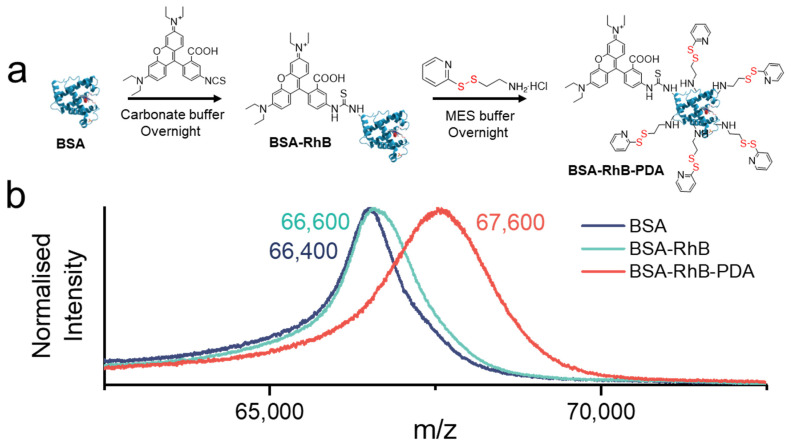
(**a**) Synthetic route of BSA modification with rhodamine B isothiocyanate (RhB) and disulfide bonds (PDA). (**b**) MALDI-TOF spectra in the [*M* + X]^+^ (X: Na or H) range of BSA, BSA-RhB, and BSA-RhB-PDA, respectively.

In order to be captured by the hydrogels, BSA was modified with RhB for imaging purposes and with PDA to contain disulfide bonds (Figure 4a). PDA containing the disulfide bonds was synthesized following an established protocol (Figure S1) [60,68]. BSA is an economical and easy-to-obtain protein that contains a large amount of carboxyl and amino groups, which is why it was selected for the modification. BSA was first modified with RhB isothiocyanate, which reacted with the amino group of BSA. After that, BSA-RhB was modified by PDA through the condensation reaction of amino and carboxyl groups using EDAC activation [69]. Both modifications were analyzed by MALDI-TOF-MS (Figure 4b). Differences in molecular weights allowed to determine the number of RhB and PDA groups per BSA biomacromolecule. The difference between BSA and BSA-RhB was about 200 Da and since one RhB weighs about 500 DA, an average of 0.4 RhB molecule was bound per protein, i.e., 40% of the BSA biomacromolecules were modified with RhB. Partial modification was supported by the shape of the MALDI-TOF spectrum of BSA-RhB as it closely overlaps with the mass spectrum of pure BSA in the lower range until 66,400 m/z (maximum for BSA), but then departs from the BSA mass spectra in the region beyond the maximum intensity (Figure 4b). Similarly, the difference between BSA-RhB and BSA-RhB-PDA was about 1000 Da and since one PDA weighs about 200 DA, an average of 5 PDA molecules were bound per protein. Both degrees of modification were considered sufficient for good imaging (0.4 eq. RhB) and high binding affinity (5 eq. PDA) of the BSA-RhB-PDA.

### 3.5. Protein Capture and Release by Hydrogel Dots in Microfluidics

**Figure 5:**
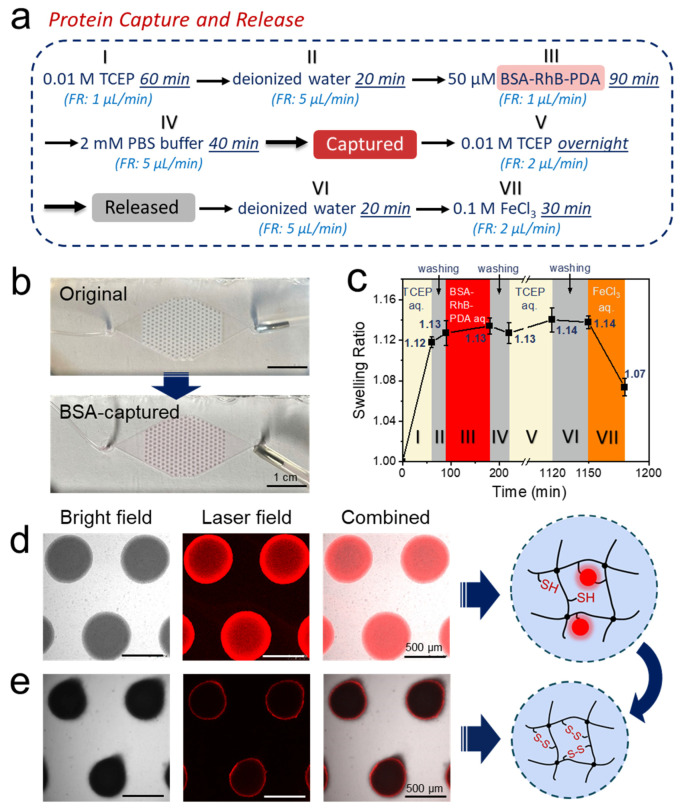
(**a**) Description of one measurement cycle to capture and release BSA-RhB-PDA, (**b**) Optical images of the original and BSA-captured hydrogel dot array in the microfluidic chip. (**c**) Swelling behavior of PNiPAAm hydrogel dots in one complete measurement cycle (**d**) Micrographs of the PNiPAAm hydrogel dots after BSA-RhB-PDA has been captured, (**e**) Micrographs of the PNiPAAm hydrogel dots after completing one whole cycle (i.e., after releasing the previously captured BSA-RhB-PDA of (**b**)). Both cartoons (**d**,**e**) refer to the composition of each hydrogel dots at the time of the measurements. All the hydrogel dots studies were performed in microfluidic chip at room temperature and observed by confocal laser fluorescence microscope under bright field, laser field and the fields combined at the height of 80 μm (channel height: 150 µm).

For microfluidic device, each hydrogel dot array contained 227 hydrogel dots of the N 2:1 gel (Figure 5b). The large number of hydrogel dots greatly increased the contact area between the hydrogel and the solution, ultimately leading to a more efficient reaction setup. The purging sequence of capturing and releasing protein is shown in Figure 5a. It should be noted that a compulsory cleaning step with a washing solution was inserted after all reaction steps to completely remove the reaction solution from the microfluidic chamber (Figure 5a,c, segments II, IV and VI) [9,59]. A low flow rate of 1 µL min^−1^ was chosen for all binding steps, a slightly higher flow rate of 2 µL min^−1^ for all unbinding steps to prevent the formation of bubbles, and the highest flow rate of 5 µL min^−1^ was used for all cleaning steps to cleave all material bound by non-specific interactions. As calculated in Table S2, the residence time of the fluid in the microfluidic chamber was 4.7 min under a flow rate of 5 μL min^−1^, and 23.6 min when 1 μL min^−1^ was applied. In order to ensure a complete reaction and thorough removal of any cleaved biomacromolecules, a longer perfusion time than the theoretical value (20–40 min for 5 μL min^−1^ and 60–90 min for 1 μL min^−1^) was used in each process step.

In the protein capture process, 0.01 M TCEP aqueous solution was first perfused at the flow rate of 1 μL min^−1^ for 60 min to break the disulfide bonds, followed by a washing step with deionized water for 20 min at the flow rate of 5 μL min^−1^ (Figure 5a, segments I and II). Afterwards, 50 μM BSA-RhB-PDA aqueous solution was perfused at the flow rate of 1 μL min^−1^ for 90 min to capture the protein, followed by a washing step with 2 mM PBS buffer (pH 7.4) for 40 min at the flow rate of 5 μL min^−1^ (Figure 5a, segments III and IV). This large excess ensured maximum binding of the modified BSA which was not quantified further. Since the protein has a higher solubility and stability in the buffer, the 2 mM PBS buffer (pH 7.4) was used to clean the chip after protein capture. As expected, the presence of BSA-RhB-PDA in the hydrogel dots could be verified with optical and fluorescence microscopy after this procedure (Figure 5b (optical), the entire process diagram is shown in Figure S8 and Figure 5d (fluorescence)). In addition to the protein capture, the swelling of the hydrogel dots was also monitored closely. Following the treatment with aqueous TCEP the hydrogel dots were swelling up to 1.12 times of the original diameter due to the breaking of disulfide bonds. (60 min in Figure 5c, segment I). It should be noted that this is the same value, the N 2:1 dots reached in the previous experiments, highlighting the reproducibility of the hydrogel dot formation process (Table 1). After removing the reducing agent with deionized water, BSA-RhB-PDA aqueous solution was administered to bind the protein to the hydrogel dots. The degree of swelling did not change over the washing and binding step, remaining at 1.13 after 220 min (Figure 5c, segment III). One BSA-RhB-PDA biomacromolecule had one or more binding sites to the hydrogel dots, depending on steric hindrance. These multiple binding sites and reformation of the disulfide bonds did hence not impact the degree of swelling of the hydrogel dots and the cross-linking density of the hydrogel dots remained unchanged.

Two controls were necessary to prove the successful capture of proteins. The first one was the same experiment, but without TCEP to break the disulfide bonds (procedure in Figure S9). As a result, no free thiol groups were available for the exchange with the disulfide bonds on the modified protein. Hence no protein was captured (Figure S10a) and the swelling ratios remained at 1.00 (Figure S11). The second control experiment did see TCEP reduction but then BSA-RhB aqueous solution was administered. Without modification by PDA, the protein did not possess the disulfide bonds required for covalent binding, resulting in a very low amount of captured protein (Figure S10b shows extremely low fluorescence intensity). Together with the controls, these experiments proved the successful thiol-disulfide exchange which enabled the protein BSA-RhB-PDA to be captured by the hydrogel dots.

In the protein release process, 0.01 M TCEP aqueous solution was purged at the flow rate of 2 μL min^−1^ overnight (Figure 5a, segment V). When TCEP was re-purged into the chip, the disulfide bonds between the hydrogel dots and the protein broke, ultimately releasing the protein. The longer time was necessary to dissociate the disulfide bonds and wash off as much BSA-RhB-PDA as possible. As a result, only few BSA-RhB-PDA remained after the release and the following washing step (fluorescence images in Figure 5e). The swelling ratio of hydrogel dots after protein release was 1.14 (after 1150 min in Figure 5c, segment V). Since there was no significant change in cross-linking density, the swelling ratio of the hydrogel dots remained almost constant (slight increase from 1.12 to 1.14) after the first purging with TCEP aqueous solution at the end of protein release (Figure 5c, segments II-VI). After cleaning with deionized water, the microfluidic chip was treated with 0.1M FeCl_3_ aqueous solution to re-oxidize thiol groups and reform disulfide bonds in the hydrogel dots (Figure 5a,c, segments VI and VII). With the increasing cross-linking densities, the swelling ratio of the hydrogel dots decreased to 1.07 (after 1180 min in Figure 5c, segment VII). Conformational changes in the hydrogel dots after breaking the disulfide bonds presumably prevented a complete shrinking to the original size.

**Figure 6:**
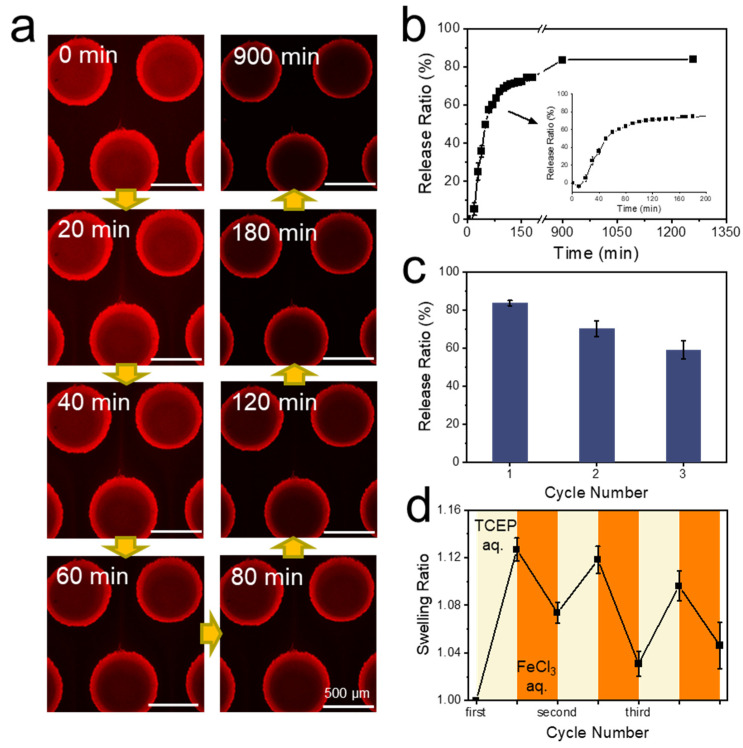
(**a**) Development of the fluorescence images of the PNiPAAm hydrogel dots over time in the microfluidic chip, during the release of BSA-RhB-PDA (image height set to 80 μm). (**b**) Release profile of BSA over time derived from the fluorescence images of Figure 6a, analyzed by ImageJ. (**c**) Release ratios of BSA captured by PNiPAAm hydrogel over three cycles. (**d**) Swelling ratios of the PNiPAAm hydrogel dots in each capture and release cycle. Every cycle consists of two data points, representing the highest swelling with broken disulfide bonds after treatment with 0.01 M TCEP and the reduced swelling after reforming these bonds with 0.1 M FeCl_3_. For (**b**–**d**) at least three hydrogel dots were tested for each experimental point to obtain reliable data.

Release profile of BSA biomacromolecules was investigated next and was followed by confocal laser fluorescence microscopy (Figure 6a). The release ratio over time (Figure 6b) was analyzed from the remaining fluorescence intensity (Figure S12). A release of 10% hence corresponded to 90% remaining fluorescence. It could be noted that the fluorescence intensity increased initially within 10 min and then dropped dramatically, which was attributed to the short increase in background noise generated by the release of the protein and the enrichment in the solution. After 3 h of release (after 180 min in Figure 6b), 74.5% of the protein had been released from the hydrogel dots. The number increased to 83.6% after 15 h (after 900 min in Figure 6b), finally reaching 84.0% after 21 h (after 1260 min in Figure 6b). Thus, the release time of the protein was fixed at 15 h in the following cyclic tests due to the very low increase in release after 15 h.

Cyclic tests of protein capture and release have been performed following the complete characterization of the first cycle. Each cycle went through the seven steps of hydrogel dots swelling, protein capture, protein release, and hydrogel dots shrinking, including all washing steps. The release ratio of protein decreased from 70.1% in the second cycle to 59.0% in the third cycle (Figure 6c). Essentially, each step resulted in a release rate of about 85% of the previous value leading to a release rate of 61% after three cycles (as 0.85^3 = 61%), which is close to the observed 59.0%. This decay was likely to continue over possible next cycles, but we did not address this point. A repeated capture and release of the device was hence possible, but declined in quality with each cycle. Swelling ratios of the hydrogel dots in cyclic tests were measured as well (Figure 6d). Every cycle consists of two data points, beginning from the first purging of 0.01 M TCEP aqueous solution and ending after the purging of 0.1 M FeCl_3_ aqueous solution. The swelling ratios of hydrogel dots under TCEP reduction decreased marginally from 1.13 to 1.10, while swelling ratios of hydrogel dots under FeCl_3_ oxidation were 1.07, 1.03, and 1.05 in the three cycles, following no pattern. It could be concluded that the swelling and shrinking of the hydrogel dots in cyclic tests showed no clear trend, lacking complete reproducibility of the first cycle. Combined with the reduced protein release ratio, the hydrogel dots exhibited a decreasing response to redox stimuli under the long-term shearing stress. However, the cyclic tests still confirmed that it was possible to capture and release protein through the disulfide bonds on the hydrogel dots in microfluidics for at least three cycles. This thiol-based capture and release of proteins in microfluidics through hydrogel dots over at least three cycles showed that our previously reported redox responsive double cross-linked hydrogels are not only an interesting concept, but are also fit for applications.

## 4. Conclusions

In summary, the PNiPAAm hydrogel dot arrays were successfully integrated into the PDMS-on-glass microfluidic device through photopolymerization. The hydrogel dot array inside the microfluidics was cross-linked by the permanent cross-linker BIS and the reversible cross-linker BAC. Cleavage or re-formation of redox-responsive disulfide bonds introduced by cross-linker BAC changed the cross-linking densities of the hydrogel dots, making them swell or shrink under redox conditions. Following rheological and compression measurements, hydrogel dots with a 2:1 ratio of BAC to BIS proved to be the mechanically most stable hydrogel dots with an appropriate degree of swelling. The thiol-disulfide exchange allowed the hydrogel dots to successfully capture and release the protein BSA modified by PDA containing disulfide bonds and dye RhB. The release ratio of protein reached 83.6% in the first cycle and proved to be reproducible on the same chip, reaching a release ratio of 59.0% after the third cycle. This selective capture and release of proteins on a microscopic scale through the redox-responsive hydrogel dots bares the advantages of minimal amount of sample and successful reusability. Thus, it has great potential for future applications as it opens up the possibility of capturing and releasing various, differently functionalized proteins, enzymes or drugs. The reported process has all prerequisites to become a lab-on-a-chip device for rapid detection and/or delivery of various (macro)molecules.

## Figures and Tables

**Table 1:** Swelling behaviors and mechanical properties of PNiPAAm hydrogel dots in microfluidics.

Hydrogel	Swelling in 0.01 M TCEP aq. ^a^	Residual Swelling in 0.1 M FeCl_3_ aq. ^b^	Percentage Reduction ^c^	Compression Strength at 50% Strain (kPa) ^d^	Elastic Modulus (Pa) ^d^
N 1:1	1.09 ± 0.007	1.06 ± 0.007	33%	5.66 ± 0.17	43 ± 5
N 1.5:1	1.10 ± 0.010	1.06 ± 0.003	40%	13.03 ± 0.60	86 ± 3
N 2:1	1.12 ± 0.014	1.07 ± 0.001	42%	15.85 ± 0.55	143 ± 3
N 3:1	1.14 ± 0.004	1.09 ± 0.003	36%	7.42 ± 0.13	53 ± 2
N 4:1	1.15 ± 0.014	1.10 ± 0.013	33%	4.55 ± 0.08	23 ± 2
N 5:1	1.16 ± 0.009	1.09 ± 0.004	44%	1.47 ± 0.14	10 ± 1

^a^ Compared to the original size after 60 min at 1 µL min^−1^ flow rate (flow rate reasoned in the main text); ^b^ compared to the original size after 30 min at 2 µL min^−1^ flow rate (flow rate reasoned in the main text); ^c^ percentage of oxidative shrinkage compared to reduction swelling; ^d^ determined at room temperature and at a 0.05 mm s^−1^ constant linear rate of compressive stress.

## Data Availability

The data presented in this study are available on request from the corresponding author.

## Supplementary Materials

The following are available online at https://www.mdpi.com/article/10.3390/polym14020267/s1. Table S1: Compositions for the synthesis of PNiPAAm hydrogels cross-linked by BAC and BIS. Table S2: Parameters and formulas applied for the calculation of the residence time of the substrates in the microfluidic device. Figure S1: Synthetic route and ^1^H NMR of PDA incl. the peak assignment. Figure S2: (a) Photomask for structuring hydrogel dots. (b) Top view of master for production of single-chamber PDMS sheet. (c) Top view (photograph) and side view (schematic) of POM mold for production of hydrogel dots. Figure S3: Hydrogel arrays prepared by photopolymerization of monomer solutions in mould with single chamber. Figure S4: Photos of the entire microfluidic setup. Figure S5: Appearances of the original, the reduced and the oxidized N 5:1 hydrogel dots. Figure S6: Swelling behaviors of PNiPAAm hydrogel dots with different mole ratios of cross-linker BAC to BIS in the microfluidic chip at different flow rates. Figure S7: (a) Frequency dependence of the loss factor (tan *δ*) for the bulk PNiPAAm hydrogels. (b) Typical compression stress–strain curves of bulk PNiPAAm hydrogels. Figure S8: Photos of the process of capturing BSA in the microfluidic chip. Figure S9: The experimental pro-cedure of both controls. Figure S10: Micrographs of the hydrogel dots in two control tests. Figure S11: (a) Optical images of the hydrogel dots in the first control test and (b) the swelling ratios of them. Figure S12: Fluorescence intensity of the PNiPAAm hydrogel dots over time in the BSA re-leasing procedure.
