# Supplementary material for: Reversible Protein Capture and Release by Redox-Responsive Hydrogel in Microfluidics
Source: Polymers (Basel). 2022 Jan 10;14(2):267. doi: 10.3390/polym14020267 (PMC8780672; doi:10.3390/polym14020267)
Supplement: Supplementary file 1 [file polymers-14-00267-s001.zip › polymers-1530519-Revised Supplementary Information.pdf]

Supplementary Information

Reversible protein capture and release by redox-responsive hydrogel in microfluidics

*Chen Jiao[a, b], Franziska Obst[c], Martin Geisler[a], Yunjiao Che[a], Andreas Richter[c], Dietmar Appelhans[a], Jens Gaitzsch[a, *] and Brigitte Voit[a, b, *]*

a Leibniz-Institut für Polymerforschung Dresden e.V., Hohe Straße 6, 01069 Dresden, Germany

b Lehrstuhl Organische Chemie der Polymere, Technische Universität Dresden, Faculty of Science, 01062, Dresden, Germany

c Institut für Halbleiter- und Mikrosystemtechnik, Technische Universität Dresden, 01187 Dresden, Germany

Table of Contents

1. Additional Methods 3

a. Design of Microfluidic Setup. 3

b. Mechanical Properties of Bulk Hydrogel. 4

c. Experimental Details on Hydrogel Production 5

2. Additional Data 6

a. ^{1}H-NMR of PDA 6

b. Photolithography 7

c. Microfluidic Setup 9

d. Characterization of the Hydrogel Dots 10

e. Capture and Release of the Protein 11

f. Residence Time of the fluid 14

3. References 14

1. Additional Methods

a. Design of Microfluidic Setup.

The polydimethylsiloxane (PDMS) sheet used to construct the PDMS-on-glass microfluidic devices was manufactured by hard and soft lithography in a standard process. First, a glass substrate was rinsed with acetone, isopropanol and deionized water. Afterwards, three resist layers (DFR, 50μm) were laminated on the glass slide and baking at 85 °C for 3 min was performed after each layer. Next, the resist was irradiated with UV light through a photomask for 90 seconds, and baked at 85°C for 40 min. Afterwards, the resist was evolved in a developer and rinser bath, and finally baked at 85°C for 1 h. The height of the produced PDMS was 161 ± 1 μm, measured by confocal microscope (Figure S2b). Subsequently, the PDMS elastomer base agent and the curing agent (Sylgard 184, Dow Corning) were mixed at a mass ratio of 10:1 (according to the supplier's instructions) and stirred for 15 min until distributed evenly. The mixture was then poured onto the PDMS master, degassed, and cured overnight at 40 °C. Finally, the PDMS sheet was peeled off from the mould and perforated to form the fluidic inlet and outlet by a biopsy punch (Kai Industries Co., Ltd, Ø: 1.5 mm). Prior to assembling the microfluidic device, the PDMS sheet was cleaned by ultrasonic treatment in isopropanol (Figure S4).

The whole microfluidic setup was composed of a syringe pump, a bubble trap, and a microfluidic device (Figure S4). The glass slide with attached hydrogel arrays was rinsed with deionized water, and dried with a cotton swab. Note that the hydrogel dots

cannot be touched during drying because they are fragile and easily moved. Then, the prepared PDMS sheet was placed onto and aligned with the glass slide to form the microfluidic device (PDMS-on-glass chip), thereby sealing the chip. The chip was covered with an aluminum frame with a transparent acrylic glass window and placed in an aluminum clip to prevent the PDMS sheet from loosening from the glass slide during the test.[1] Through the transparent window, the fluid flow inside the microfluidic chip can be observed. Finally, the microfluidic pump (LA-100, LANDGRAF HLL, Germany), the bubble trap (LVF-KBT-L, Darwin Microfluidics, Paris, France) and the microfluidic device were connected in series through PTFE tubes and Cannulas (TechLab, Germany). The bubble trap was used to remove bubbles in the solution. Based on the test, the response time (that is, the time required to fill the microfluidic chip and reach the outlet tube) was about 8 min at a pumping flow rate of 5 μL min^{-1}. Parameters and formulas applied for the calculation of the residence time of the substrates in the microfluidic device are shown in Table S2.

b. Mechanical Properties of Bulk Hydrogel.

The cylinder bulk hydrogels (diameter ca. 7 mm, height ca. 7 mm) used to measure the mechanical properties were prepared following the same method as reported. The mechanical properties of the as-prepared bulk hydrogel were determined on an ARES-G2 rheometer (TA Instruments, New Castle, GB) equipped with 25 mm parallel plates and an evaporation blocker. The instrument was calibrated for zero gap before the test. For rheological test, storage modulus (G') and loss modulus (G'') were measured under room temperature (T = 25 °C) and a fixed oscillation strain (γ = 1%) over a frequency

range from 0.1 to 100 rad s^{-1}. The distance between the two plates is constant. For compression test, a sample was loaded between two parallel plates and subjected to a compressive stress at a constant linear rate of 0.05 mm s^{-1} until a determined 50% strain was reached. The elastic modulus (E) is determined by the slope of the linear compression curve with strain below 20%.

c. Experimental Details on Hydrogel Production

Table S1. Compositions for the synthesis of PNiPAAm hydrogels cross-linked by BAC and BIS.

Hydrogel	Cross-linker (2 mol%)		NiPAAm (12.5 wt%)	Photoinitiator LAP	Solvent	
	BAC	BIS			Water	Ethanol
**N 1:1**	21.61 mg, 1.00 mol%	12.80 mg, 1.00 mol%	930.8 mg, 8.3 mmol	15.87 mg, 0.65 mol%	5.90 mL	0.71 mL
**N 1.5:1**	25.93 mg, 1.20 mol%	10.24 mg, 0.80 mol%				
**N 2:1**	28.74 mg, 1.33 mol%	8.57 mg, 0.67 mol%				
**N 3:1**	32.42 mg, 1.50 mol%	6.40 mg, 0.50 mol%			5.50 mL	1.22 mL
**N 4:1**	34.58 mg, 1.60 mol%	5.12 mg, 0.40 mol%				
**N 5:1**	36.09 mg, 1.67 mol%	4.22 mg, 0.33 mol%				

2. Additional Data

a. ^{1}H-NMR of PDA

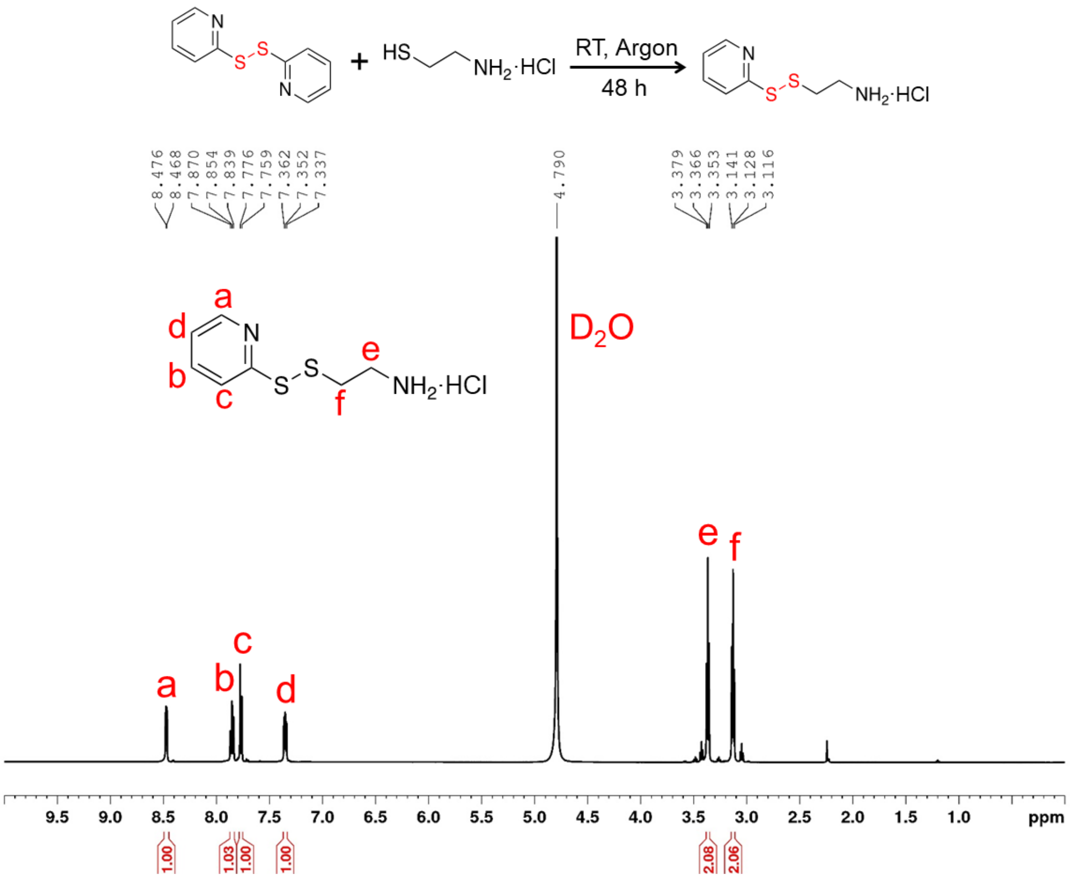

Figure S1. Synthetic route and ^{1}H NMR of PDA incl. the peak assignment.

b. Photolithography

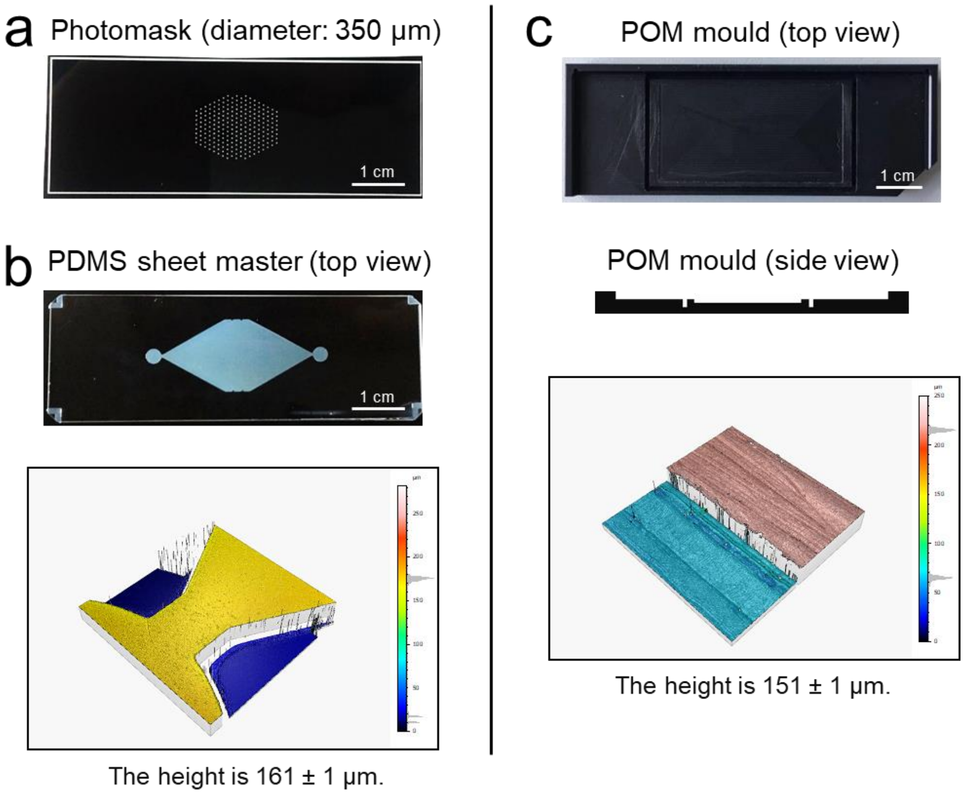

Figure S2. (a) Photomask for structuring hydrogel dots. Size of the mask: 7.6 × 2.7 cm^2. Diameter of the dots: 350 µm. (b) Top view of master for production of single-chamber PDMS sheet. The height is 161 µm, determined by confocal microscope. (c) Top view (photograph) and side view (schematic) of POM mould for production of hydrogel dots. The height is determined by confocal microscope.

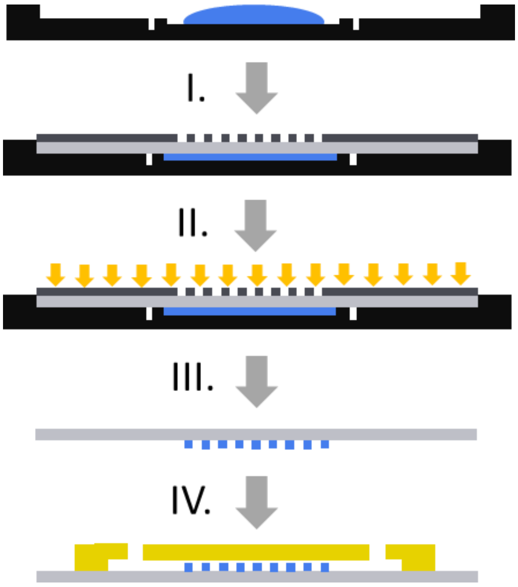

Figure S3. Hydrogel arrays prepared by photopolymerization of monomer solutions in mould with single chamber, adapted from published method [10]. (I) Alignment of the glass slide and photomask on the POM mold filled with the monomer solution, (II) UV-irradiation, (III) hydrogel arrays on glass slide, (IV) alignment of the PDMS chip on the glass slide to seal the microfluidic chip.

c. Microfluidic Setup

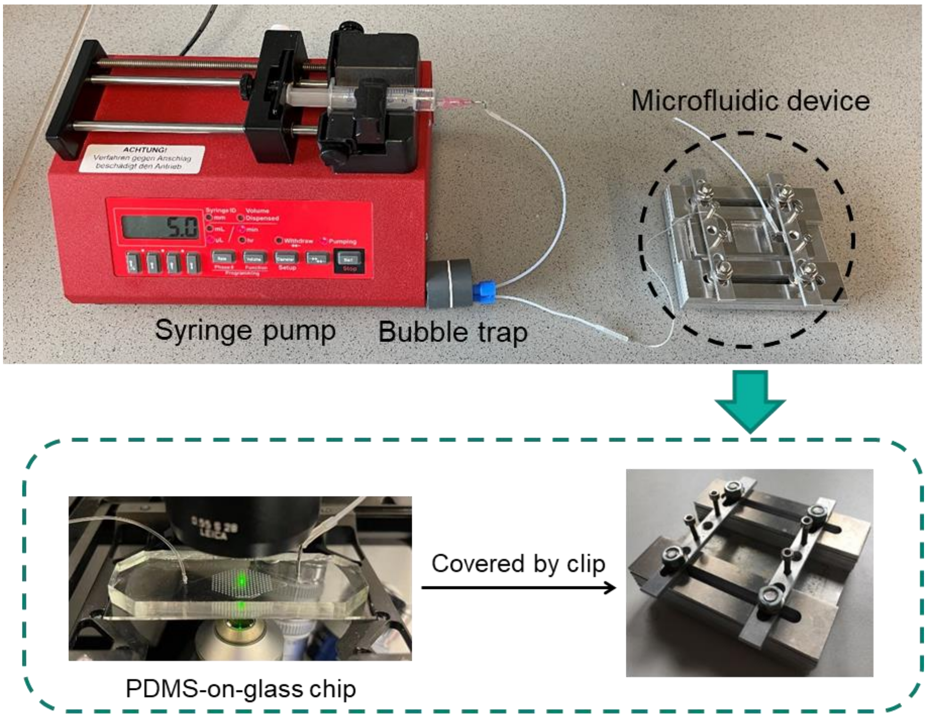

Figure S4. Photos of the entire microfluidic setup. A syringe, controlled by a microfluidic pump, a bubble trap and a microfluidic device are connected in series through tubes. The microfluidic device is composed of a PDMS-on-glass chip placed in an aluminum clip.

d. Characterization of the Hydrogel Dots

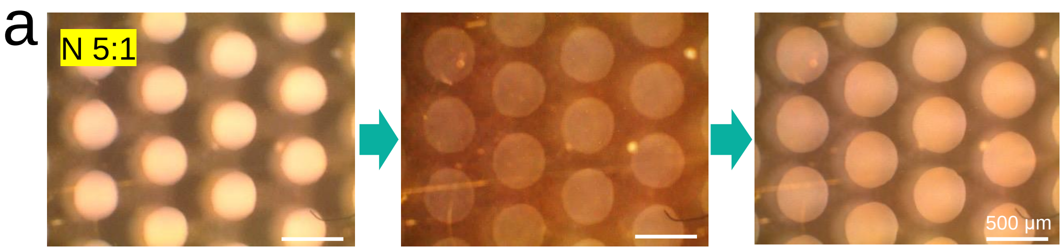

Figure S5. Appearances of the original (left), the reduced (middle) and the oxidized (right) N 5:1 hydrogel dots.

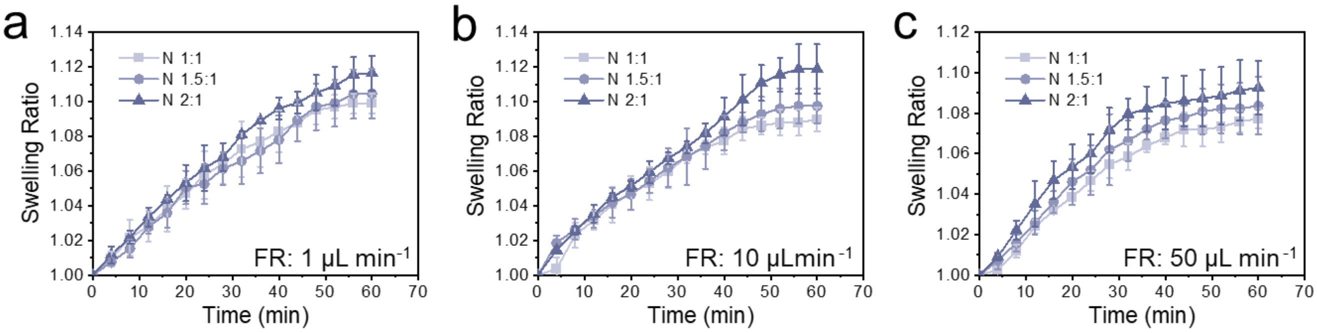

Figure S6. Swelling behaviors of PNiPAAm hydrogel dots with different mole ratios of cross-linker BAC to BIS (BAC:BIS = 1:1, 1.5:1, 2:1) in the microfluidic chip under 0.01 M TCEP aqueous solution perfusing at a flow rate of (a) 1 μL min^{-1}, (b) 10 μL min^{-1} and (c) 50 μL min^{-1}. At least three hydrogel dots were tested for each experimental point to obtain reliable data.

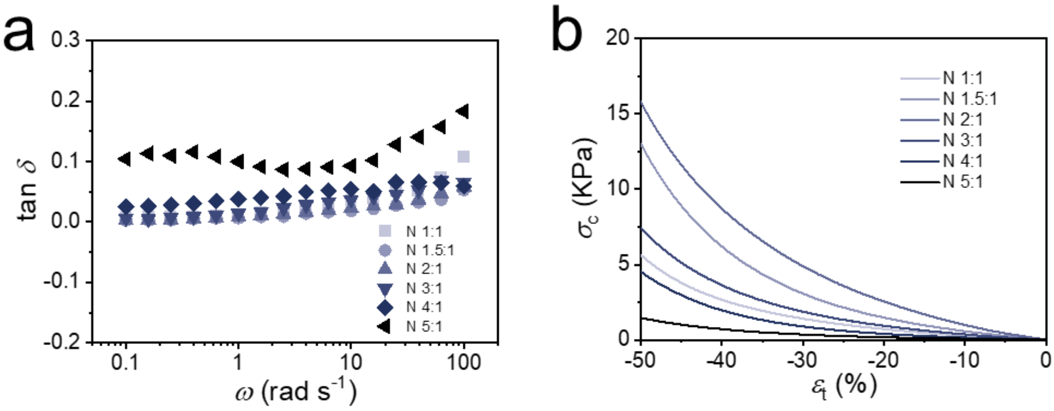

Figure S7. (a) Frequency dependence of the loss factor (tan δ) for the bulk PNiPAAm hydrogels (from N 1:1 to N 5:1 hydrogel) at a fixed temperature (T = 25 °C) and strain (γ = 1%). (b) Typical compression stress–strain (σ_c–ε_t) curves of bulk PNiPAAm hydrogels (from N 1:1 to N 5:1 hydrogel).

e. Capture and Release of the Protein

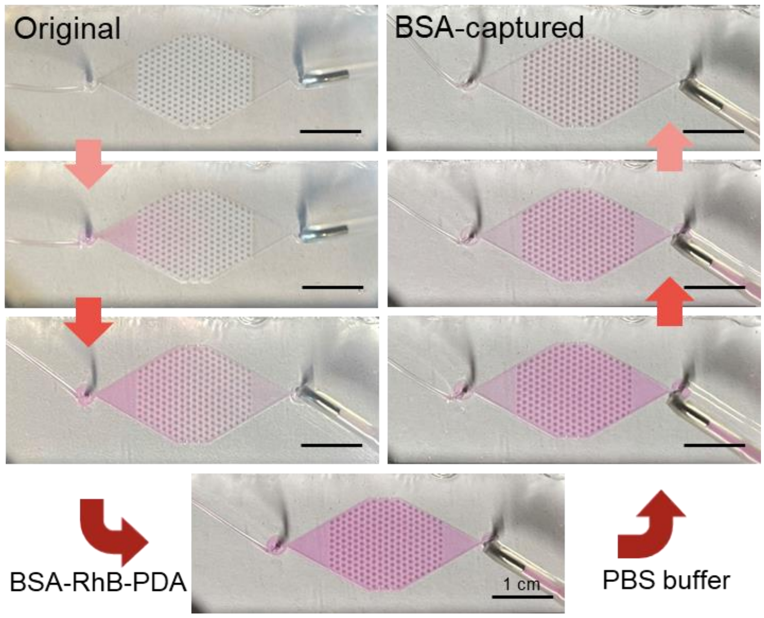

Figure S8. Photos show the process of capturing BSA in the perfusion of 50 μM BSA-RhB-PDA aqueous solution.

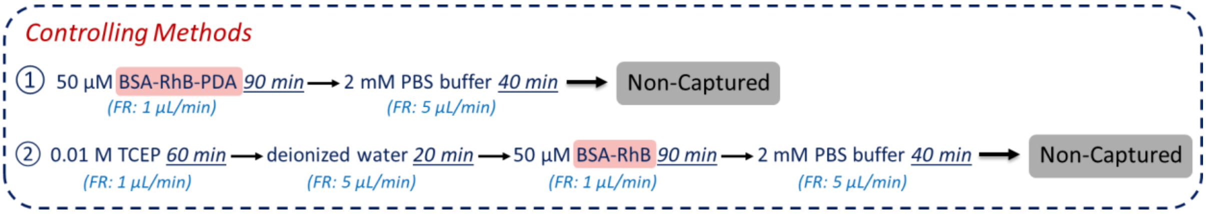

Figure S9. The experimental procedure of both controls (1 – no TCEP and 2 – no PDA on BSA), leading to no captured protein in the microfluidic device.

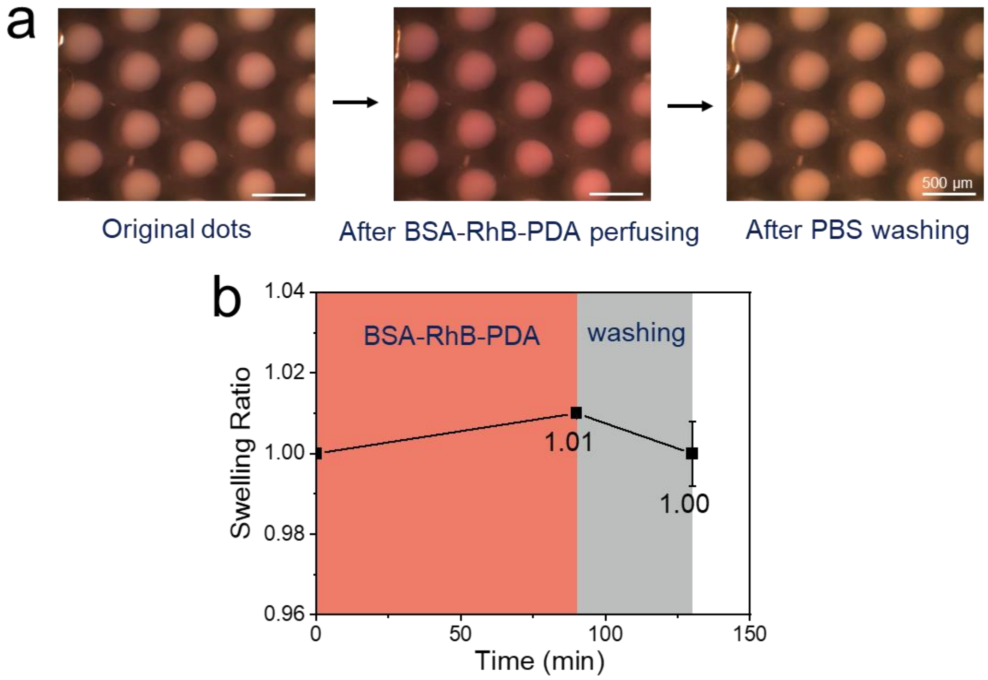

Figure S11. (a) Optical images of the hydrogel dots in the first control test (see Figure S8), the appearances of original PNiPAAm hydrogel dots, the one after injecting the BSA-RhB-PDA solution (without injecting TCEP first to break the disulfide bonds) and the one after continuous perfusing PBS buffer to wash. (b) The swelling ratios of the PNiPAAm hydrogel dots in this control test. At least three specimens were tested for each experimental point to obtain reliable data.

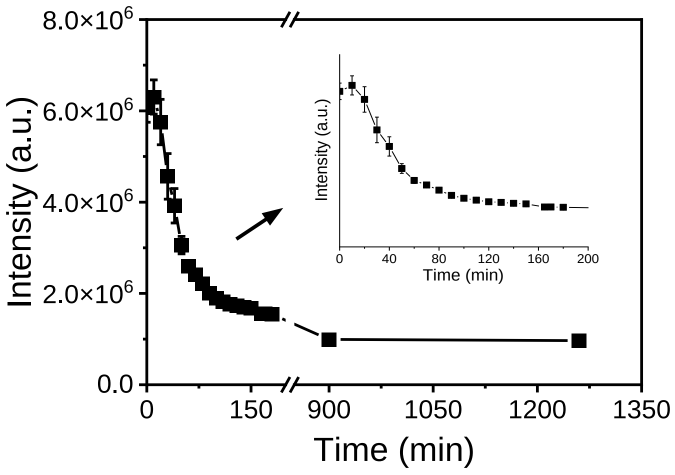

Figure S12. Fluorescence intensity of the PNiPAAm hydrogel dots over time in the BSA releasing procedure, which was measured by confocal laser fluorescence microscope at the height of 80 μm and analyzed by ImageJ. At least three specimens were tested for each experimental point to obtain reliable data.

f. Residence Time of the fluid

Table S2. Parameters and formulas applied for the calculation of the residence time of the substrates in the microfluidic device.

Geometry of the Microfluidic Device			
Amount of hydrogel dots	n_{dot}	227	Defined by photomask
Radius of hydrogel dots	r_{dot}	0.175 mm	Defined by photomask (diameter: 350 µm)
Height of hydrogel dots	h_{dot}	0.15 mm	Defined by POM mold (depth: 150 µm)
Height of microfluidic chamber hchamber	$h_{chamber}$	0.16 mm	Defined by PDMS sheet (chamber depth: 160 µm)
Area of hydrogel dot array	$a_{hydrogel\ array}$	168 mm²	Defined by photomask
Volume of the microfluidic chamber	$V_{chamber}$	26.88 µL	$V_{chamber} = a_{hydrogel\ array} \times h_{chamber}$
Volume of n hydrogel dots	V_{dots}	3.30 µL	$V_{dots} = \pi\ (r_{dot})^2 \times h_{dot}$
Fluid volume in microfluidic chamber	V_{fluid}	23.58 µL	$V_{fluid} = V_{chamber} - V_{dots}$
Residence time of fluid in microfluidic chamber (flow rate: 10 µL min^{-1})	$t_{R\ (10\ \mu L\ min^{-1})}$	2.4 min	$t_R = V_{fluid} \times (\text{flow rate})^{-1}$
Residence time of fluid in microfluidic chamber (flow rate: 5 µL min^{-1})	$t_{R\ (5\ \mu L\ min^{-1})}$	4.7 min	$t_R = V_{fluid} \times (\text{flow rate})^{-1}$
Residence time of fluid in microfluidic chamber (flow rate: 1 µL min^{-1})	$t_{R\ (1\ \mu L\ min^{-1})}$	23.6 min	$t_R = V_{fluid} \times (\text{flow rate})^{-1}$

3. References

10. Obst, F.; Simon, D.; Mehner, P.J.; Neubauer, J.W.; Beck, A.; Stroyuk, O.; Richter, A.; Voit, B.; Appelhans, D. One-step photo-structuring of multiple hydrogel arrays for compartmentalized enzyme reactions in microfluidic devices. *React. Chem. Eng.* **2019**, *4*, 2141–2155.
